# Hepatitis B Vaccination Among Medical Students

**DOI:** 10.4103/0970-0218.39254

**Published:** 2008-01

**Authors:** A Vinodhkumaradithyaa, M Srinivasan, RA Sankarasubramanian, A Uma, I Ananthalakshmi, P Thirumalaikolundusubramanian, P Kanagasundaram

**Affiliations:** Institute of Microbiology, Madurai Medical College, Madurai, India. E-mail: vinodkumar4u2@gmail.com; 1 Institute of Internal Medicine, Madras Medical College, Chennai, India.; 2 Department of Community Medicine, Madurai Medical College, Madurai, Tamilnadu, India.

Sir,

The incidence of liver disease associated with the hepatitis B virus (HBV) is a world-wide public health problem. The disease is transmitted by perinatal, parenteral, sexual routes.(1) Healthcare workers, particularly surgeons, pathologists, dentists and physicians working in hemodialysis and oncology units, are at a higher risk of contracting HBV infection via minor skin cuts and accidental needle punctures.(2) Since proper vaccination can prevent HBV infection, complete hepatitis B vaccination is necessary for medical and paramedical students.

The present study was carried out to observe the attitude and practice of medical students towards hepatitis B vaccination and find out if they completed all three doses and, if not, elicit the reasons for incomplete vaccination.

The study group consisted of medical students admitted in the year 2000 and 2001. After giving a brief introduction on the study, pre-tested, self-administered, anonymous questionnaires were distributed with reference to hepatitis B immunization history and reasons for non-compliance among those who had not completed three doses. The questionnaires were distributed in a single day in June 2005 in order to avoid sharing of information. The data were analysed statistically using Chi-square test.

Out of the 250 questionnaires circulated, 217 (M = 130, F = 87) were returned, and the response rate was 86.8%. Among them, only 29 (22.3%) boys and 21 (24.1%) girls maintained their vaccination record. Nineteen (8.7%) (9 boys, 10 girls) students had already completed three doses and received booster dose on admission. Unfortunately, without vaccination against HBV, 12 (9.2%) boys and 3 (3.4%) girls produced certificates from qualified and registered medical practitioners. Among the rest (183), the numbers that received the first, second and third dose of hepatitis B vaccination were 109 (100%), 62 (56.9%) and 33 (30.3%) boys, respectively, and 74 (100%), 41 (55.4%) and 31 (41.9%) girls, respectively. Complete coverage was significantly (*P* < 0.05) more among girls. The reasons for non-compliance were many and are mentioned in Table 1. Some dominant reasons were forgetfulness (38.8%), no reminder (35.8%), lack of knowledge on hepatitis B infection (23.1%), lack of compulsion (20.1%), etc.

**Table 1:** Reasons for non-compliance of hepatitis B vaccination

No.	Reasons*	Boys, *N* = 88	Girls, *N* = 46	Total, *N* = 134	
				
		No.	No.	No.	%
1	I have forgotten	25	27	52	38.8
2	None reminded me	36	12	48	35.8
3	I have not been informed about hepatitis B infection, complications and prevention	22	9	31	23.1
4	No one compelled me	15	12	27	20.1
5	I leave things to the Almighty	10	9	19	14.2
6	My parents did not encourage me	8	2	10	7.4
7	I thought it was not required now	5	4	9	6.7
8	I do not believe in vaccination	3	2	5	3.7
9	I have to spend money for vaccine	3	3	6	4.5
10	I am not involved in any procedures with patients	2	2	4	2.9
11	Others	No one motivated us I thought one dose was enough			

* Total number exceeded the participants in view of multiple answers

Medical students are susceptible to HBV infection during their exposure to clinical cases and different procedures. Hence, medical students were advised to have vaccination against HBV before coming to clinical side, as they acquire good immune response with immune memory.(3) Three doses of vaccination are provided free of charge to all the students (both day scholars and hostelites) during the time of admission. They were provided with a vaccination card indicating the schedule for subsequent doses.

For want of follow-up, reminding, monitoring and supervising systems, 119 did not complete vaccination. Hence, it is suggested that the students and their parents/family members who come on the day of admission should be educated on HBV infection and motivated for successful and effective vaccination. The process of vaccination may be completed with the help of student council. The study also highlights the need for a national policy in countries where hepatitis B vaccination to health care personnel is not made mandatory. Limitations of this study include non-availability of vaccination record in many and recall bias in addition to non-screening of their blood for hepatitis B surface antigen and antibodies too.
